# Aging phenomena in non-crosslinked polyolefin blend cable insulation material: Electrical treeing and thermal aging

**DOI:** 10.3389/fchem.2022.903986

**Published:** 2022-11-08

**Authors:** Li Lunzhi, Gao Jinghui, Zhong Lisheng, Zhang Kai, Zhao Xiaohan

**Affiliations:** ^1^ School of Electronic and Control Engineering, Chang’an University, Xi’an, China; ^2^ State Key Laboratory of Electrical Insulation and Power Equipment, Xi’an Jiaotong University, Xi’an, China

**Keywords:** cable insulation, non-crosslinked, polyolefin blend, electrical treeing, thermal aging

## Abstract

Non-crosslinked polyolefin blends have become a favorable alternative material to crosslinked polyethylene (XLPE) cable insulation owing to their low power consumption in the production process and good recyclability at the end of service life. Although studies on non-crosslinked materials have achieved significant results, the electrical and thermal aging properties of these materials undeniably need extensive research attention and systematic exploration. Aging performance is directly related to the lifetime and reliability of cables. In this study, the electrical treeing and thermal aging phenomena of 70 wt.% linear low-density polyethylene (LLDPE) and 30 wt.% high-density polyethylene (HDPE) blends (abbreviated as 70L–30H) were studied and compared with those of XLPE by investigating the microstructural feature, electrical treeing behavior, and mechanical performance during thermal aging. Electrical treeing tests show that 70L–30H blends exhibited smaller treeing dimensions and lower electrical tree growth rates than those of XLPE. Thermal aging tests exhibit that the mechanical property degradation of 70L–30H blends is less than that of XLPE under the same aging time. Through differential scanning calorimetry analysis and microstructure observation, the 70L–30H blend shows higher melting temperature, thicker lamellae, and higher crystallinity with a uniform and fine spherulite structure, which are responsible for good anti-aging performance. This study indicates that the blends exhibit better electrical and thermal aging resistance than XLPE, which provides a performance guarantee for its further application in the non-crosslinked cable system.

## Introduction

Recently, the development strategy of “low-carbon and environmental protection” around the world has attracted progressively more attention. The power industry, as an important part of the traditional energy field, inevitably carries out technological innovation. This technological advancement can lead to the improvement of electrical materials and power equipment in terms of environmental friendliness. XLPE has become the preferred material for extruded cable insulation for decades, attributed to its excellent mechanical and electrical properties. However, its disadvantages such as high energy consumption, a long manufacturing cycle, and difficulty in recycling after cable abandonment around the production and preparation process have aroused serious research concerns at the scientific and engineering levels. Thus, searching for new low-energy consumption and recyclable non-crosslinked cable materials has become an urgent task for the cable industry.

Polymer blending has been proven to be an effective and significantly important approach for material modification (Fortelny et al., 2019; Aliotta et al., 2020). Thermoplastic polymer blends, which are a new cable insulation material and an effective alternative to XLPE, exhibit lower energy consumption in the production process and better recyclability at the end of service life (Stevens et al., 2011a; Stevens et al., 2011b; Fairhurst et al., 2012). Hosier et al. (1997) and Hosier et al. (2000) found that the blends of low-density polyethylene (LDPE) and high-density polyethylene (HDPE) could form space filling texture spherulite, which could enhance the electrical strength and inhibit the growth of the electric tree (Green et al., 2011). Moreover, the mini-cables based on the blends mentioned earlier demonstrated higher DC breakdown strength than XLPE mini-cables (Green et al., 2013). Besides, polypropylene (PP)-based blends have been extensively investigated. Propylene–ethylene copolymer (PEC) blends can improve low-temperature impact toughness (Zhang et al., 2017). The DC test of PP-based blend insulated mini-cables also confirms their better electrical strength than that of XLPE (Green et al., 2015). Zhou et al. (2015) and Zhou et al. (2019) successively studied the properties of different PP-based blends and copolymers and further conducted a high-temperature–dependent research on PP-based thermoplastic polypropylene (Pengfei et al., 2019). In our previous studies, the modification of linear LDPE (LLDPE) as a matrix resin by blending it with HDPE was reported (Li et al., 2015a; Li et al., 2015b; Zhang et al., 2015). The results indicated that the blends containing 70 wt.% LLDPE and 30 wt.% HDPE could form a special molecular chain aggregation state and lead to significant improvements in mechanical and electrical properties (Zhang et al., 2016; Li et al., 2018a; Zhang et al., 2019), even at high temperatures (Li et al., 2018b).

Although non-crosslinked blend insulation materials have been extensively investigated, most of them are based on the short-term mechanical and electrical properties of the blend. However, a study on the aging properties of these blends has never been carried out. Notably, the aging properties of insulation materials are directly related to the long-term stability and reliability of the cable system. In this study, the electrical and thermal aging characteristics of LLDPE/HDPE blends were studied. Besides, the influence mechanism for the aging behavior of LLDPE/HDPE blends was discussed through crystallization and morphology characterization.

## Experimental setup

### Sample preparation

Two commercial polyethylene materials, namely, LLDPE and HDPE [the density and melt index of LLDPE are 0.923 g cm^−3^ and 0.2 g/10 min (190°C, 2.16 kg), respectively, and those of HDPE are 0.945 g cm^−3^ and 0.75 g/10 min (190°C, 2.16 kg)] with the weight ratio of 70:30, were selected as the matrix resin. The blends were blended using a twin-screw extruder (the screw diameter d = 41.1 mm, the bottom diameter d = 27.1 mm, and the draw ratio L/D = 40:1) with different section temperatures of 150, 160, 160, 170, 170, 180, 180, and 160°C, successively, which was followed by granulation after cooling. The granules were further hot-pressed to different thickness and shape requirements for corresponding tests at 170°C and 10 MPa for 10 min. For comparative analysis, a commercial XLPE granular material provided by the manufacturer, which was used for 110 kV cable insulation, was also prepared. The XLPE samples were first pre-crosslinked at 4.5 MPa and 120°C for 5 min and then formed by hot pressing at 10 MPa and 150°C for 15 min.

### Morphological structure characterization

Differential scanning calorimetry (DSC) was used to determine the melting behavior of each specimen. A heat flow differential scanning calorimeter (DSC q2000; American TA) was used in this study. The mass of the sample to be analyzed was 5–10 mg. The melting temperature for all specimens was in the range of 20–140°C with a heating rate of 10°C min^−1^. During DSC tests, nitrogen with a flow rate of 50 ml min^−1^ was introduced as a protection.

The samples for microscopic morphology observation were first etched at 70°C for 1 h in a chemical reagent (95 wt.% concentrated sulfuric acid/5 wt.% potassium permanganate solution), which was followed by spray-gold treatment before observation by scanning electron microscopy (SEM). The SEM was carried out using a scanning electron microscope (Hitachi S2700) with an electronic acceleration voltage of 20 kV.

### Electrical treeing tests

The samples for electrical treeing tests were cut into pieces (25 mm × 20 mm × 5 mm). Then, the high-voltage needle electrode (1.0 mm in diameter at the bottom and 5 ± 1 μm in radius at the tip with a 30° point angle) was inserted into the specimen so that the needle tip was 3 mm away from the ground electrode. The entire setup was then immersed in silicone oil to avoid surface flashover. An AC voltage of 20 kV and 50 Hz (in root mean square) was applied between needle plate electrodes and maintained for 20 min at a normal atmospheric temperature, that is, 50 and 70°C.

### Thermal aging tests

Thermogravimetric analysis (TGA) was performed using a thermogravimetric analyzer (Mettler Toledo, Zurich, Switzerland). The temperature for TGA for all materials was in the range of 50–600°C, with three different heating rates of 5, 10, and 20 K min^−1^. The sample mass was between 5 and 10 mg. Then, the data were analyzed using the Origin software, and the activation energy of the thermal oxygen aging reaction was calculated using the Flynn–Wall–Ozawa method.

Owing to the limitation of the melting point of blends, the temperature for thermal aging selected in this study was 125°C (slightly lower than the melting temperature of the blends). After hot pressing, the samples were hung in the constant temperature oven and kept in their natural vertical state. The aging environment of all materials was kept consistent by opening the external circulation system of the oven. One sample was taken out every 30 days for visual inspection and tensile tests.

### Mechanical tests

The variation of mechanical modulus with a temperature range (25–140°C, 1 K min^−1^) was measured by dynamic mechanical thermal analysis (DMA; Mettler Toledo, Zurich, Switzerland). The size of samples fixed with a dual cantilever beam fixture was 50 mm × 10 mm × 5 mm and was tested under a sinusoidal force of 1 N and 1 Hz.

The dumbbell-type samples for tensile tests were prepared using a vulcanizing machine at 175°C and 10 atm for 5 min, and the thickness of the samples was about 1 mm. Tensile tests were carried out *via* a tensile testing machine (CMT450) with an extension rate of 50 mm min^−1^ at room temperature.

For all tests, an annealing treatment was performed for 24 h at 70°C on the samples to relax the internal stress before the tests.

## Results and discussion

**FIGURE 1:**
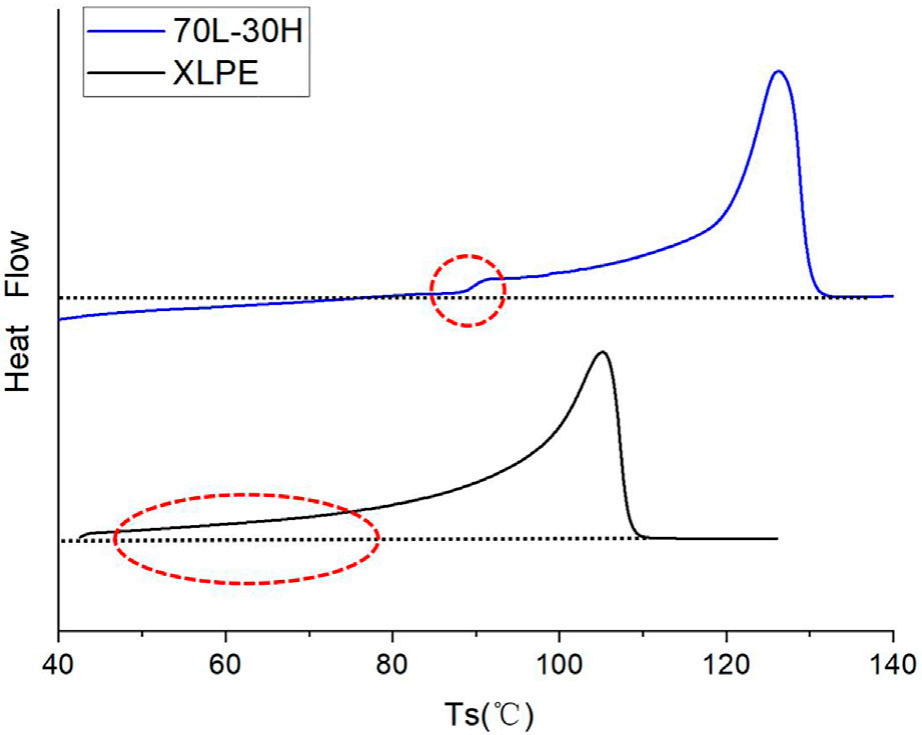
DSC melting traces of various samples.

Figure 1 shows the DSC results, demonstrating that 70L–30H blends exhibit a higher melting temperature than XLPE. Moreover, the DSC curve of blends shows a single peak as a superposition of the melting peaks of the two components, which indicates that the melting temperatures of the two base materials are almost similar. This is conducive to the formation of a eutectic structure between the two materials, which was discussed in our previous research. From the perspective of crystallization kinetics, the higher melting temperature corresponds to thicker lamellae and more perfect crystallization (Sun et al., 2011). It was found that XLPE begins to melt at a low temperature (below 70°C), corresponding to some microcrystals (their crystallization behavior is limited due to the chemical crosslinking bond). Analysis of DSC curves and sample masses indicates that the melting enthalpies of blends and XLPE are 112.198 and 94.913 J g^−1^, respectively. Then, the crystallinity of the two materials can be calculated using the following equation:

$$x_{c}=\Delta H_{m}/\Delta H_{m\infty }\times 100\%,$$ (1)

where *ΔH*_m_ is the melting enthalpy per unit mass, and *ΔH*_m∞_ (287.3 J/g) denotes the melting enthalpy of 100% crystallinity for PE (Mirabella and Bafna, 2002). Through calculation, the crystallinity of the blend and XLPE is 39.1% and 33.0%, respectively.

These results correspond to our previous successive self-nucleation and annealing (SSA) analysis. It is well known that the normal operating temperature of a power cable is about 70–90°C. However, the melting phenomenon of XLPE at about 70°C definitely affects the electrical and mechanical performance of the cable under operating temperature, thus affecting the long-term stability and reliability.

**FIGURE 2:**
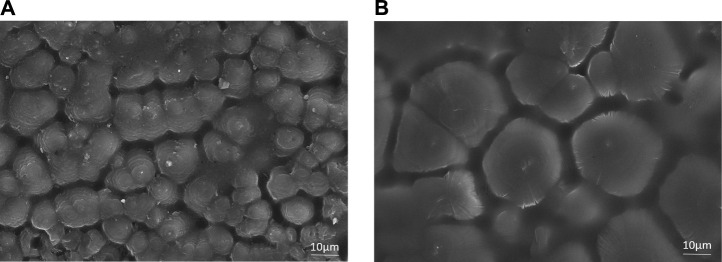
SEM images showing the spherulite form of **(A)** 70L–30H blends and **(B)** XLPE.

From the SEM image of Figure 2, it can be seen that the spherulite structure of XLPE is obviously different from blends, with a significantly larger spherulite diameter of 20 μm. It is because that the crystallization temperature of XLPE is lower; this is due to the high branching degree of XLPE base material LDPE on the one hand and the influence of the crosslinking bond on the other hand. According to the study of crystallization kinetics of polyethylene, the molecular chain with a high branching degree has a lower crystallization rate under the same undercooling, which means that XLPE has a lower nucleation rate and crystal growth rate (Hoffman and Weeks, 1962; Patel, 2012). Therefore, XLPE does not have enough nuclei to form more spherulites but can only form large spherulites at low temperatures, leading to a significant increase in the grain boundary and amorphous region. The spherulite diameter of 70L–30H blends is about 10 μm. Also, the spherulite size has better uniformity, forming a more uniform and fine spherulite structure. It is the micro reason why blends have excellent electrical and mechanical properties, which has also been verified in our previous works.

**FIGURE 3:**
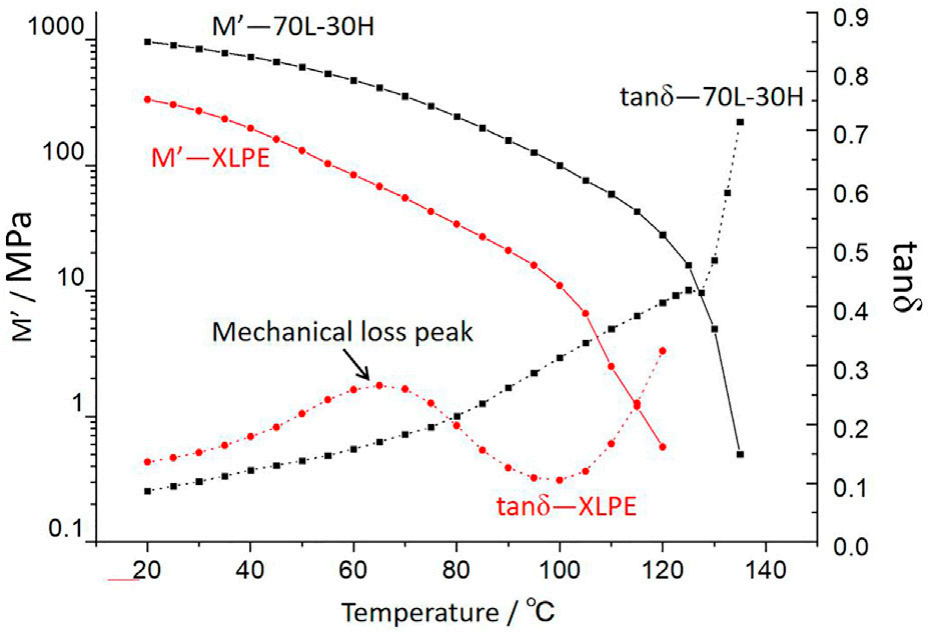
Temperature spectrum for DMA of 70L–30H and XLPE.

Figure 3 exhibits the DMA, showing that the module of blends and XLPE decreases gradually with the temperature. The curves exhibit a cliff-like shape near the melting point, corresponding to an exponential rise in the loss factor. The mechanical loss of XLPE shows an obvious peak at about 60–70°C, which is different from that of blends. The mechanical loss peak corresponds to the melting of the crystal, which is consistent with the results of the DSC analysis.

**FIGURE 4:**
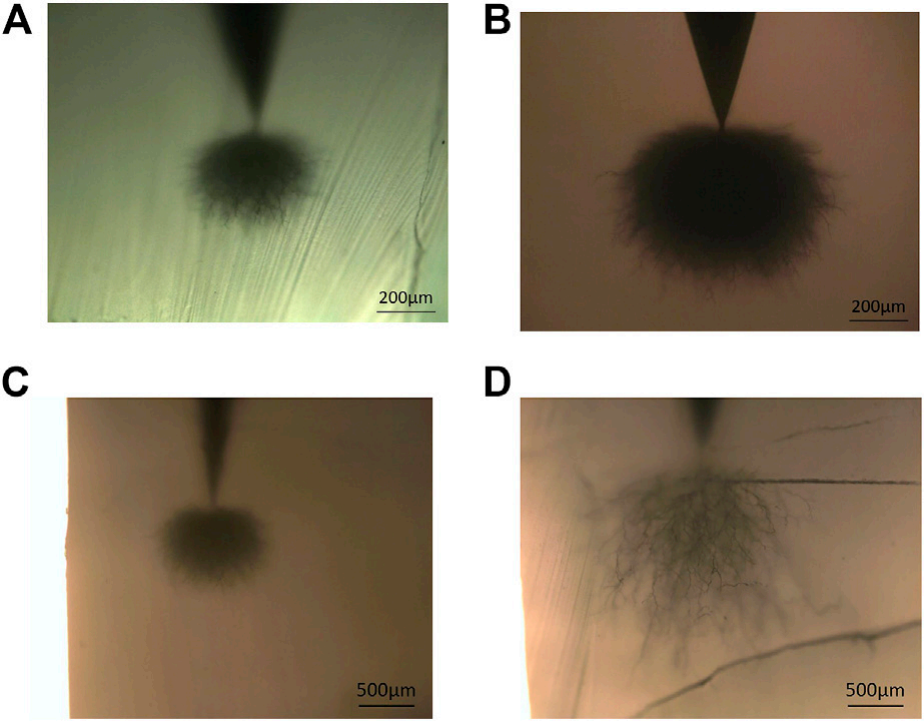
Optical microscopic observation after electrical treeing tests for **(A)** 70L–30H at room temperature, **(B)** XLPE at room temperature, **(C)** 70L–30H at 50°C, and **(D)** XLPE at 50°C.

Figure 4 shows the growth results of electric treeing. Clearly, both 70L–30L blends and XLPE exhibit the growth of dense jungle-like electrical trees after electrical treeing aging at room temperature; however, the size of electrical trees grown in XLPE is significantly larger than that of blends. At 50°C, the electrical tree size of the two materials is apparently larger than that at room temperature. Nonetheless, 70L–30H blends still show an obvious jungle-like electrical tree with a lower vertical growth length (average 834 μm), while XLPE shows a dendritic electrical tree with a larger vertical growth length (average 2,026 μm). At 70°C, both materials broke down in a short time and failed to reach the predetermined withstand voltage time of 20 min. The average breakdown times of XLPE and 70L–30H are 2 min 54 s and 13 min 26 s, respectively, indicating that the electrical tree resistance of 70L–30H is still better than that of XLPE at this temperature.

Based on the distance between the needle tip and the ground electrode, the average growth rate of an electric tree at 70°C was calculated by utilizing the average breakdown time. Table 1 presents the comparison of the average electrical tree growth rate between the two materials at room temperature, 50°C, and 70°C. Clearly, the electrical tree growth rate at 70°C is significantly increased by more than ten times than that at room temperature. Among them, the ratio of the XLPE growth rate at 70°C to that at room temperature is higher, while the corresponding ratio of 70L–30H is lower. It is demonstrated that the electrical tree resistance of XLPE decreases faster with the increase in temperature. In contrast, the electrical tree resistance of the 70L–30H blend decreases slightly with the increase in temperature, which represents better temperature stability.

**TABLE 1:** Average electrical treeing growth rate of XLPE and 70L–30H blends at various temperatures (μm min^−1^).

Sample	Room temperature	50°C	70°C	70°C/R
70L–30H	18.6	41.7	223.3	12.0
XLPE	29.1	101.3	1,034.5	35.5

The initiation and growth of the electrical tree are due to the electro-stress mechanical crack caused by the gradual fatigue of the medium under the impact of accumulated energy, which then leads to the fracture of molecular chains, indicating that the growth characteristics of the electrical tree are closely related to the mechanical properties of the medium. Zeller and Schneider (1984) reported that the growth of the electrical tree is required to overcome the energy of internal surface tension and yield strength. As a typical semi-crystalline polymer, PE exhibits higher surface tension and yield strength in its crystalline region due to the more regular structure; in contrast, the amorphous region has lower mechanical properties. Ding and Varlow (2002) studied and calculated the dynamic model of electric microcrack growth for semi-crystalline polymers and found that the electric tree growth frequency K can be expressed as follows:

$$K=\left(\frac{kT}{h}\right)\exp\left(\frac{\alpha C_{0}\pi \varepsilon E^{2}-U_{0}}{kT}\right),$$ (2)

where *k* and *h* are the Boltzmann and Planck constants, respectively; *T* is the absolute temperature, *α C*_0_ is the volume activated by the local electric field; *U*_0_ is the activation energy of the breakdown process; *ε* is the dielectric permittivity; and *E* is the electrical field strength.

Chi Xiaohong found that the breakdown activation energy of the crystalline region near the needle electrode was much higher than that of the amorphous region through simulation research based on the growth dynamics of electrically induced microcracks by Ding and Varlow (2002) and Chi et al. (2014). Based on the abovementioned studies, it is not difficult to infer that the electrical tree generally grows along the amorphous region in PE.

Through the aforementioned electrical tree growth kinetic mechanism, 70L–30H blends showed better electrical tree resistance, which can be explained based on their crystallinity and morphological structure. After filling HDPE, the blends formed thicker lamellae with higher crystallinity, leading to stronger internal mechanical strength and an increase in the energy required for electro-stress mechanical cracks to be overcome, which inhibited the growth of electrical treeing. According to our previous research, the thick lamellae formed by the blend material get filled in the amorphous region and grain boundary of LLDPE, so that the electric tree has to bypass the thick lamellae to grow in its development process, which hinders the development of the electric tree along the direction of the electric field. On the contrary, more regular spherulite morphology and uniform phase structure can reduce the concentration of impurities and amorphous regions in blends, inhibit electric field distortion, and reduce local field strength. Therefore, the blend exhibits excellent electrical tree resistance. However, the internal mechanical strength of XLPE is lower due to its low crystallinity and thin lamellae. Moreover, there are more amorphous regions in XLPE than in blends, and larger spherulites lead to the concentration of amorphous regions and impurities. Therefore, XLPE shows the largest electric tree growth length and poor electric tree resistance.

With the increase in temperature, the polymer gradually undergoes thermal expansion and microcrystalline melting, which leads to the increase in the free volume and the decrease in the mechanical modulus. Thus, the growth rate of the electrical tree increases with the increase in temperature (Ding and Varlow, 2002; Chi et al., 2014). The analysis indicates that some thin lamellae of XLPE begin to melt at about 70°C, resulting in a large decrease in its crystallinity and a rapid increase in the amorphous region. This leads to the increase in the growth rate of electrical treeing of XLPE more than that at normal temperature and is more sensitive to temperature. 70L–30H has high crystallinity and a high content of thick lamellae; thus, only a few crystallites melt at 70°C, which limits the increase in the activation volume of submicroscopic electric treeing. Moreover, the thicker lamellae that inhibit electrical treeing have higher melting temperatures and can still maintain their lamellar morphology at 70°C, which can maintain the function of electrical tree retardancy.

**FIGURE 5:**
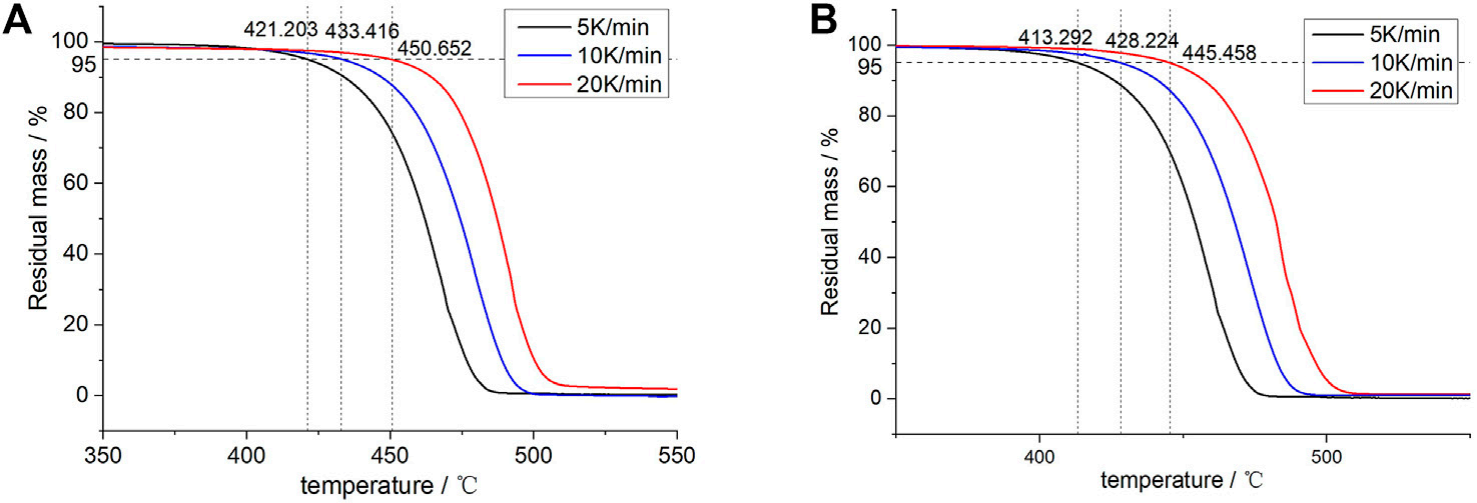
TGA curves of **(A)** 70L–30H and **(B)** XLPE under different heating rates and temperature points corresponding to a 5% weightlessness rate.

Figure 5 shows the TGA curves of blends and XLPE. Through the Arrhenius formula of chemical reaction, the activation energy, heating rate, and inverse thermodynamic temperature of a material satisfy the following equation (Ozawa, 1965; Flynn and Wall, 1966):

$$\log\beta =0.4567\left(-\frac{E_{a}}{RT}\right)+\log A,$$ (3)

where *R* is the universal gas constant, 8.314 kJ mol^−1^ K^−1^.

**FIGURE 6:**
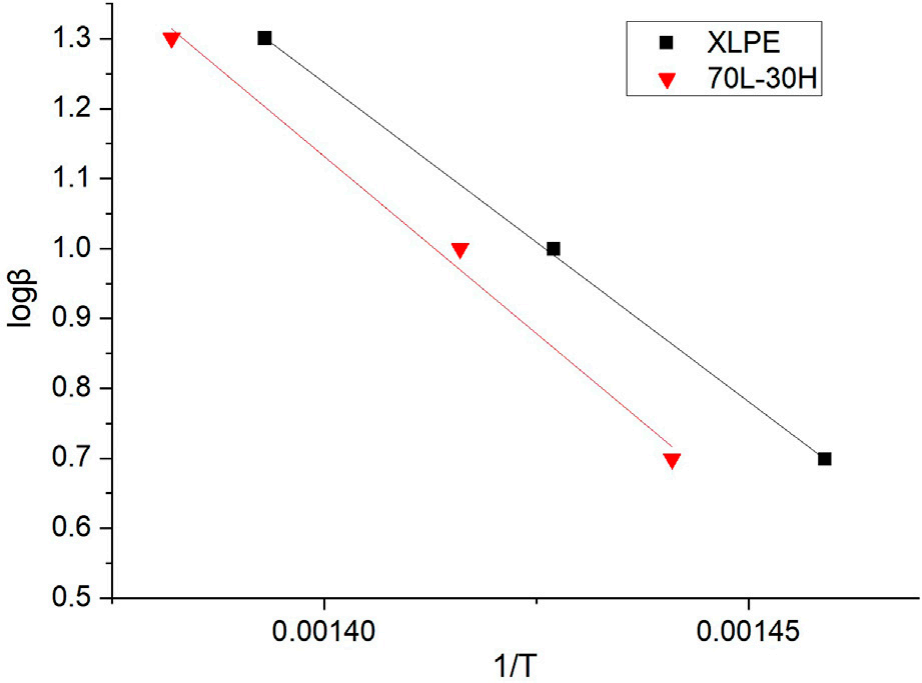
Fitting line between logβ and 1/T of 70L–30H and XLPE.

The temperature points of the material were considered corresponding to the weightlessness rate of 5% under different heating rates. For the 70L–30H blends, the temperature points obtained in the 5, 10, and 20 K min^−1^ heating curves are 421.203, 433.416, and 450.652°C, respectively, and the corresponding thermodynamic temperatures are 694.203, 706.416, and 723.652 K. Similarly, the corresponding thermodynamic temperatures of XLPE are 686.292, 701.224, and 717.458 K. The logβ and inverse thermodynamic temperatures of 1/T corresponding to the different heating rates of the two materials are plotted in Figure 6 and then linearly fitted. The fitting linear equation of the 70L–30H blends is

y=−10125x+14.85. (4)

The fitting linear equation of XLPE is

y=−9122x+13.59. (5)

From Eq. 1, the slope of the straight line k = 0.4567 (−*E*a/*R*). By substituting the fitted linear slope, the activation energy of the thermal oxygen aging reaction was calculated. The activation energy of the thermo-oxidative reaction of the 70L–30H blends is *E*a = 184.23 kJ⋅mol^−1^, and the activation energy of the thermo-oxidative reaction of XLPE is *E*a = 165.58 kJ mol^−1^. Clearly, the 70L–30H blends exhibit higher activation energy of the thermo-oxidative reaction than XLPE, which indicates that the blends require more energy for the thermo-oxidative reaction at the same temperature than XLPE, exhibiting better thermal aging resistance.

**FIGURE 7:**
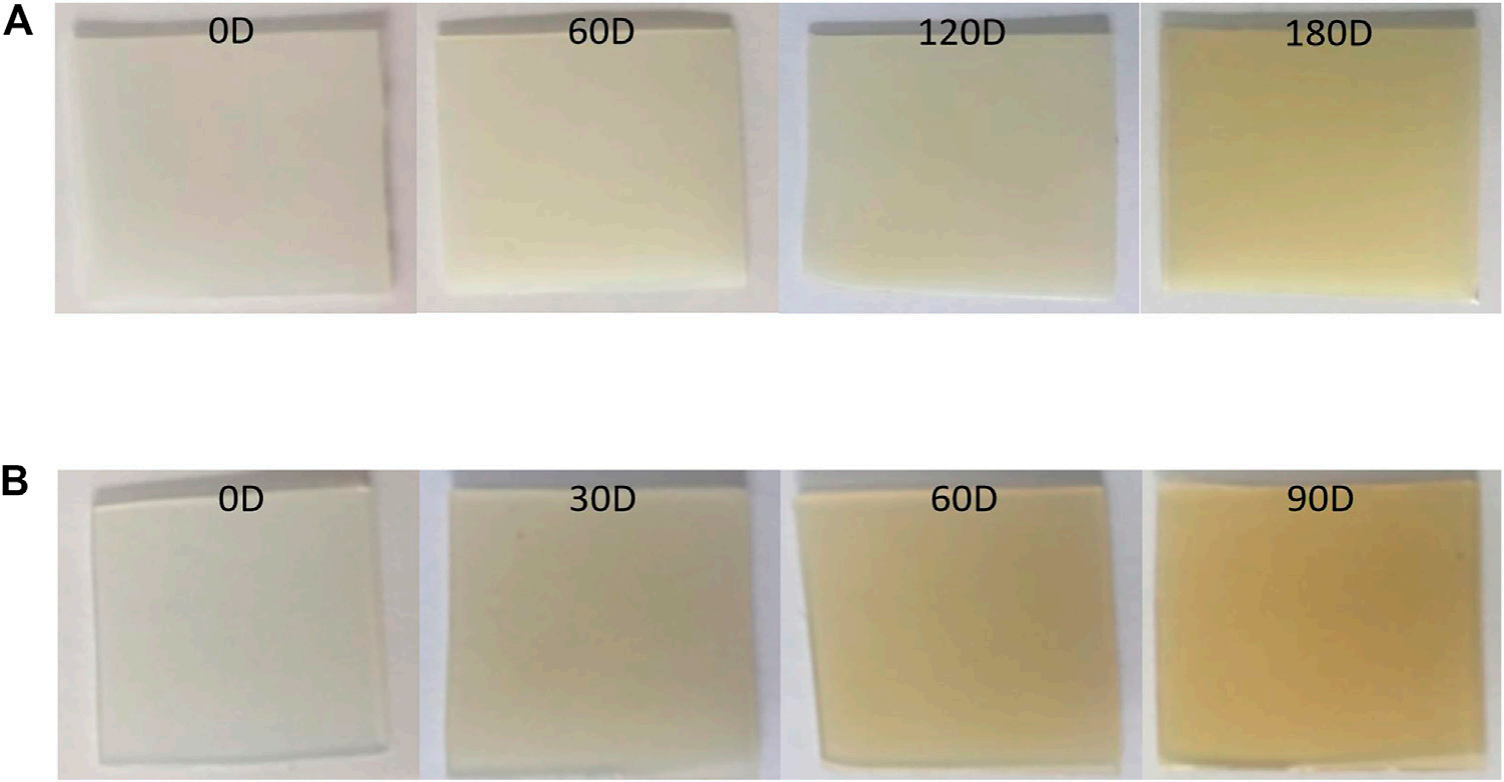
Changes in the appearance of two materials with aging time: **(A)** 70L–30H and **(B)** XLPE.

Figure 7 shows the changes in the appearance of 70L–30H and XLPE after several aging days. Figure 7 demonstrates that with the increase in the thermal aging time, both materials undergo a color change at different degrees. The color of 70L–30H blends deepens at a slower rate, showing slight yellowing from about 60 days, and an obvious yellowing phenomenon occurs at 180 days. However, the XLPE exhibits obvious yellowing from about 30 days and turns into brownish yellow at 90 days, indicating that XLPE reacts more violently in the thermal aging process, with a deeper aging degree. This is consistent with the results of the activation energy calculations reported earlier.

Table 2 lists the mechanical property changes of 70L–30H blends and XLPE with aging time through tensile tests. In the process of thermal aging, the thermal oxidation reaction is accompanied by the chain scission, leading to the deterioration of the mechanical properties of materials. The mechanical properties of the blends and XLPE did not drop significantly at the beginning of aging (0–60 days). There was even an increase in the modulus of elasticity. On the one hand, it illustrates that the antioxidant added to the material inhibits the thermal aging reaction at the early stage of aging. On the other hand, it is also related to the recrystallization of the polymer at high temperatures (Boukezzi et al., 2008; Li et al., 2021). With the increase in aging time, the aging degree of the two materials intensifies. The macromolecular chain gets broken due to splitting, and the molecular chain length decreases, which weakens the entanglement force between the molecular chains, resulting in a decrease in tensile strength. The deterioration trend for the mechanical properties of blends is slower than that of XLPE, indicating that they have better thermal aging resistance than XLPE. In fact, we did not add any stabilizer in the preparation of the blends. The base material of XLPE is directly provided by the manufacturer, which contains a certain amount of stabilizer, but the specific content is unknown due to trade secrets.

**TABLE 2:** Variation of mechanical properties for each material with thermal aging time.

Sample	Aging time/d	Elastic modulus/MPa	Tensile strength/MPa
70L–30H	0	449.71	31.12
	30	455.29 (101.2%)	29.78 (95.7%)
	60	459.32 (102.1%)	28.91 (92.9%)
	90	461.20 (102.6%)	28.83 (92.6%)
	120	457.25 (101.7%)	27.43 (88.1%)
	150	451.46 (100.4%)	26.31 (84.5%)
	180	435.82 (96.9%)	26.20 (84.2%)
	210	408.21 (90.8%)	25.51 (82.0%)
XLPE	0	145.13	23.72
	30	147.27 (101.5%)	22.98 (96.9%)
	60	150.67 (103.8%)	22.18 (93.5%)
	90	143.28 (98.7%)	21.14 (89.1%)
	120	137.61 (94.8%)	19.23 (81.1%)

**FIGURE 8:**
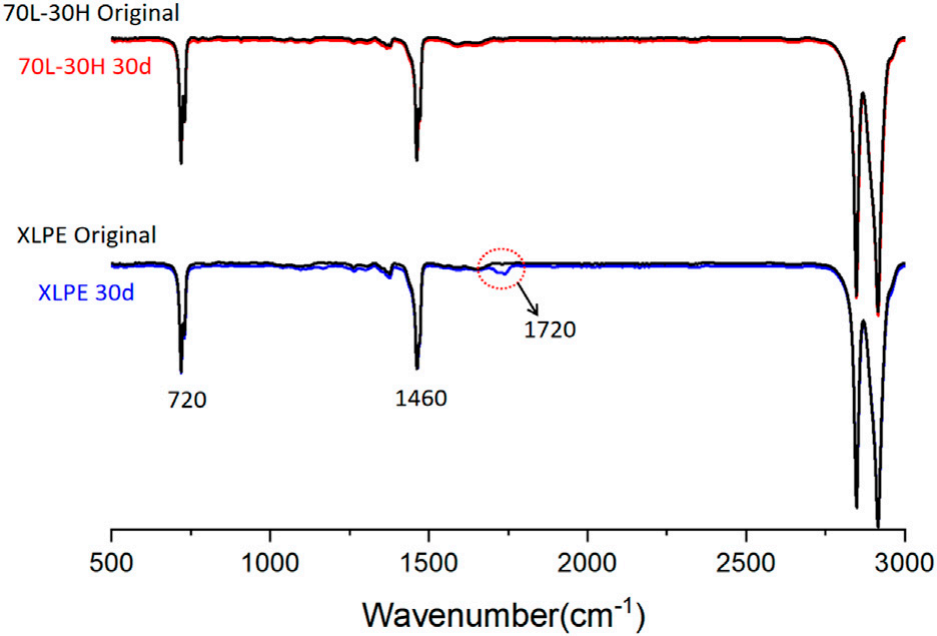
FTIR spectra of 30d thermal aged blends and XLPE.

The FTIR spectra of blends and XLPE without aging and after aging for 30 days are shown in Figure 8. It can be seen from the figure that the main absorption peaks of the unaged blends and XLPE are in the bands with corresponding wavenumber of 720, 1460 and 2800–3000 cm^−1^. After 30 days of aging, the FTIR spectra of 70 L–30 H blends basically coincided with the unaged absorption curve, and there was no unique carbonyl absorption peak of polyethylene after aging. However, a new absorption peak which is the characteristic peak of carbonyl stretching vibration corresponding to wavenumber 1720 cm^−1^ appeared in XLPE after aging, with the absorption peak intensity of wavenumber at 720 and 1460 cm^−1^ decreases slightly. The above results illustrate that after aging for 30 days, the blends did not exhibit obvious thermo oxidative products, while XLPE produced obvious thermo oxidative carbonyl products through chain scission. It indicates that 70 L–30 H blends have better thermal oxidative aging resistance than XLPE, which is consistent with the degree of appearance color change and the change of mechanical properties mentioned above.

The previous experimental results indicate that the blend has better thermal aging resistance than XLPE, its activation energy of the thermal aging reaction is higher than that of XLPE, and the rate of performance degradation is also lower than that of XLPE at the same aging days. This phenomenon can be analyzed from the composition of molecular chains and the phase structure of the blends and XLPE.

1) Molecular chain structure. According to the mechanism of thermal aging decomposition, molecular chains with high linearity do not easily undergo degradation under high temperatures to form active chains with free radicals, while PE molecular chains with a large number of branched side chains easily undergo thermal degradation reaction, resulting in the fracture of side chains and branched chains (Roy et al., 2008; Roy et al., 2009). The molecular chain linearity of LLDPE and HDPE used as base materials in this study was found to be high. Therefore, they do not easily undergo degradation under the action of heat and oxygen. The base material of XLPE is LDPE with a high degree of branching. Thus, the molecular chain contains a large number of branched chains and side chains, which are prone to thermal degradation reaction. Although many weak keys are strengthened through crosslinking, the results of DSC and SSA fractional crystallization reported in our previous studies indicate that a large number of highly branched molecular chains of XLPE still crystallize at low temperatures. Therefore, compared with XLPE, the molecular chains of blends are less prone to thermal degradation.
2) Morphological structure. According to the analysis of the results of SSA fractional crystallization, compared with the blends, XLPE shows low crystallinity and imperfect wafer, resulting in a large number of amorphous regions. Oxygen molecules diffuse more easily in the amorphous region than in the crystalline region. At 125°C, the crystalline regions in XLPE get basically completely melted, and a large number of amorphous regions become channels for oxygen to spread into the material, increasing the oxygen concentration inside the material. Although the XLPE maintains its shape because its crosslinked bonds restrict the flow of molecular chains, this type of reticular pore is too large to limit the entry of tiny oxygen molecules into the material. The increase in oxygen concentration causes an increase in the thermal aging reaction rate and acceleration of the aging process of the material. Owing to the homogeneous phase structure and the high melting point of the blends, the distribution of amorphous regions is reduced. Therefore, oxygen molecules do not easily diffuse into the materials, which thus leads to the inhibition of the internal thermal aging reaction rate of the materials.

## Conclusion

In this study, the electrical and thermal aging characteristics of LLDPE/HDPE blends were studied. Moreover, the influence mechanism for the aging behavior of LLDPE/HDPE blends was discussed through crystallization and morphology characterization.

The results of electrical treeing tests at different temperatures and the thermal aging tests indicate that the blends exhibit better electrical and thermal aging resistance than XLPE. On the one hand, it benefits from the more regular molecular chain structure of the blends, which can lead to a higher melting point and higher crystallinity. On the other hand, the blends can form a more uniform and finer spherulite structure and phase structure, which can inhibit the growth of the electrical tree and thermo-oxidative reaction morphologically. In conclusion, this study demonstrates that the blends are also provided with superiority in long-term stability and reliability.

Combined with our previous results, it can be seen that the blends can exhibit more excellent properties (including electrical, mechanical, and anti-aging properties at high temperatures) than XLPE in the temperature range below its melting point (the melting point is about 126°C). In order to make it really be used in the cable system, it will be a crucial breakthrough to improve its working temperature. This is also the focus of our future work.

## Data Availability

The raw data supporting the conclusion of this article will be made available by the authors, without undue reservation.
